# The effect of charge and albumin on cellular uptake of supramolecular polymer nanostructures†

**DOI:** 10.1039/d3tb02631k

**Published:** 2024-04-09

**Authors:** Jiankang Song, Peter-Paul K.H. Fransen, Maarten H. Bakker, Sjors P.W. Wijnands, Jingyi Huang, Shuaiqi Guo, Patricia Y. W. Dankers

**Affiliations:** a Institute for Complex Molecular Systems, Eindhoven University of Technology PO Box 513 5600 MB The Netherlands P.Y.W.Dankers@tue.nl; b Department of Biomedical Engineering, Laboratory for Cell and Tissue Engineering, Eindhoven University of Technology PO Box 513 5600 MB The Netherlands; c Department of Biomedical Engineering, Laboratory of Chemical Biology, Eindhoven University of Technology PO Box 513 5600 MB The Netherlands

## Abstract

Intracellular delivery of functional biomolecules by using supramolecular polymer nanostructures has gained significant interest. Here, various charged supramolecular ureido-pyrimidinone (UPy)-aggregates were designed and formulated *via* a simple “mix-and-match” method. The cellular internalization of these UPy-aggregates in the presence or absence of serum proteins by phagocytic and non-phagocytic cells, *i.e.*, THP-1 derived macrophages and immortalized human kidney cells (HK-2 cells), was systematically investigated. In the presence of serum proteins the UPy-aggregates were taken up by both types of cells irrespective of the charge properties of the UPy-aggregates, and the UPy-aggregates co-localized with mitochondria of the cells. In the absence of serum proteins only cationic UPy-aggregates could be effectively internalized by THP-1 derived macrophages, and the internalized UPy-aggregates either co-localized with mitochondria or displayed as vesicular structures. While the cationic UPy-aggregates were hardly internalized by HK-2 cells and could only bind to the membrane of HK-2 cells. With adding and increasing the amount of serum albumin in the cell culture medium, the cationic UPy-aggregates were gradually taken up by HK-2 cells without anchoring on the cell membranes. It is proposed that the serum albumin regulates the cellular internalization of UPy-aggregates. These results provide fundamental insights for the fabrication of supramolecular polymer nanostructures for intracellular delivery of therapeutics.

## Abstract 2

Bovine serum albumin (BSA) regulates cellular internalization of charged ureido-pyrimidinone (UPy)-assembly. The BSA-bond UPy-assembly can be internalized and co-localizes with mitochondria. Without BSA, the UPy-assembly anchors to the cell membrane.

## Graphical abstract

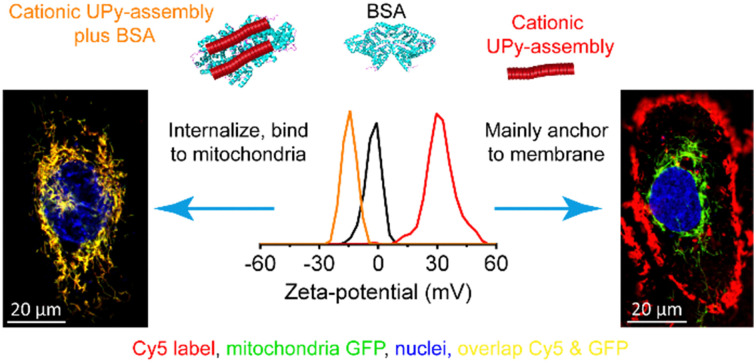

## Introduction

Efficient and effective intracellular delivery of biomolecules and exogenous compounds for advanced cell-based therapies, regenerative medicine, gene editing and fundamental biology remain to be challenged.^1–4^ To tackle this challenge, numerous synthetic nanocarriers have been developed based on inorganic nanomaterials, polymers and lipids.^2,5–7^ Additionally, targeting ligands, cell penetrating peptides and other functional molecules have been incorporated to achieve selectivity.^2,5–8^ Among these nanocarriers, various types of supramolecular nanostructures^9–22^ have gained significant interest. These nanostructures are composed of tunable molecular recognition motifs which can self-assemble through dynamic and reversible non-covalent interactions.^23,24^ These inherent characteristics allow for delicate control of the composition and physicochemical properties of the supramolecular nanostructures on a molecular level to achieve efficient delivery outcomes.^25^

Despite the promising potential of using nanocarriers for intracellular delivery, these platforms confront a complex biological barrier which severely limits their therapeutic outcomes upon systemic or local administration.^1,7^ This barrier begins with non-specific adherence of a mixture of plasma proteins including serum albumin onto their surfaces,^7,26–30^ followed by clearance of the opsonized nanocarriers by the mononuclear phagocyte system which mainly consists of resident macrophages.^31^ In addition, this protein binding alters the overall functional and physicochemical properties of the nanocarriers and thus dramatically influences their entry into targeting cells.^26,32–38^ Therefore, it is crucial to evaluate the internalization of the nanocarriers by both phagocytic and targeting cells in the presence or absence of serum proteins to provide fundamental information for developing new intracellular delivery therapies.

Although the influence of serum proteins on the cellular uptake of conventional nanocarriers has been indicated from a large number of studies,^26,29,32,34–37^ this influence on supramolecular nanostructures has not yet been investigated in detail. Besides serum proteins, the surface charge of the nanocarriers is a key parameter known to influence their cellular internalization even though the internalization procedure is not fully understood.^35,37–41^ Moreover, the influence of the charge on the cellular uptake of nanocarriers is cell-type dependent, *e.g.*, phagocytic *vs.* non-phagocytic cells.^38,39,42^ It is reported that the internalization of a self-assembled supramolecular scaffold based on 1,3,5-benzenetricarboxamide amphiphiles was either charge or receptor dominated.^15^ However, a possible difference of the influence of serum proteins on cellular internalization by either phagocytic or non-phagocytic cells was not investigated.

**Fig. 1:**
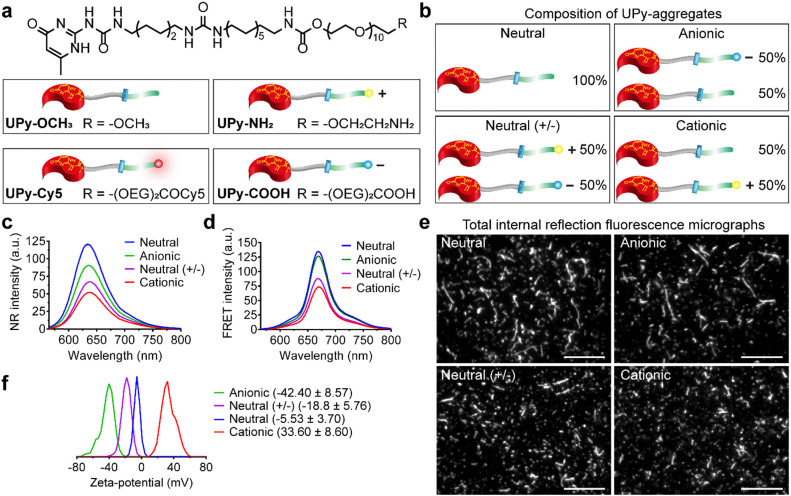
Chemical structures of the ureido-pyrimidinone (UPy)-monomers and characterizations of the various UPy-aggregates. (a) Chemical structures of the UPy-monomers with different functional groups. (b) Composition and illustration of the UPy-aggregates with different charge properties. (c) Nile red (NR) measurement for the confirmation of the assemblies of UPy-aggregates in PBS with the formation of lateral hydrophobic pockets, where the NR could be encapsulated and emitted intensive fluorescent signals. (d) Fluorescence resonance energy transfer (FRET) measurement with NR/Cy5 pair to confirm the encapsulation of fluorescent reporter UPy–Cy5 into the UPy-aggregates in PBS. (e) Total internal reflection fluorescence microscope (TIRF) of the various UPy-aggregates in PBS, the morphology of these aggregates were examined with Cy5 fluorophore. Scale bars represent 10 μm. (f) Zeta-potential measurements of the various UPy-aggregates (50 μM) in 5 mM HEPES buffer at pH 7.4, the transition of the charge properties of the UPy-aggregates from anionic to cationic has been confirmed.

Therefore, the aim of the current study was to systematically investigate cellular uptake of various charged supramolecular polymer nanostructures (1) with the presence of serum proteins to mimic the complex *in vivo* administration environment and (2) for both phagocytic and non-phagocytic cells to explore the dominate factor that determines cellular uptake. It was hypothesized that serum proteins rule over charge on cellular internalization, and that serum albumin dominates the uptake process. To this end, an amphiphilic molecule based on quadruple hydrogen-bonding ureido-pyrimidinone (UPy) supramolecular moiety,^43–45^ connected to an alkyl spacer containing an urea functionality, linked *via* an urethane group to hydrophilic oligo(ethylene glycol) (OEG) was selected as the base monomer (Fig. 1(a)). The chemical structure of the UPy-monomers *i.e.*, length of alkyl spacer and PEG densities, was optimized to achieve robust UPy-aggregates formation (unpublished data). This monomer has been shown to assemble into stable fibrous aggregates owing to the stacking of hydrogen-bonded UPy-dimers in the lateral direction, assisted by additional hydrogen-bonding of the urea functionalities and possibly the urethane groups between the alkyl and OEG part. The modularity of this supramolecular polymer system enables for diverse functionalization of monomers to achieve multifunctional UPy-aggregates in a “mix-and-match” fashion.^9,10,25^ Previously, our group has shown to be able to successfully complex RNAi therapeutics with such cationic UPy-aggregates for cellular internalization.^9,46^ In the current study, the UPy-monomers were functionalized with different end groups to obtain a supramolecular toolbox for the fabrication of four types of UPy-aggregates with various charge properties, *i.e.*, **neutral**, **anionic**, **neutral (+/−)** and **cationic** (Fig. 1(a) and (b)). To systematically investigate the influence of serum proteins and charge properties on cellular internalization, the uptake of these UPy-aggregates by THP-1 derived macrophages and immortalized human kidney cells (HK-2 cells) in the presence or absence of fetal bovine serum (FBS) were performed. Secondly, the location of the UPy-aggregates inside the cells after being incubated with cells for a certain period was probed. Finally, to test the regulatory role of bovine serum albumin (BSA) on cellular uptake, BSA was added into the cell culture medium containing UPy-monomers and subsequent internalization of the formed UPy-aggregates was monitored.

## Results and discussion

### Characterization of the UPy-aggregates

Supramolecular monomers were designed and synthesized to assemble in aqueous medium in a modular manner. To obtain functional UPy-aggregates with different charge properties, the UPy-monomers were end-functionalized with various charged groups, *i.e.*, non-charged methoxy, cationic amine and anionic carboxylate groups (Fig. 1(a)). As reporter molecules, fluorophore Cy5 or fluorescein was grafted onto the UPy-molecule to help monitor the cellular uptake of the UPy-aggregates (Fig. 1(a)). The UPy-aggregates were prepared *via* a “mix-and-match” method. With this method, the critical aggregation concentration of the UPy-monomers was determined at approximately 1 μM (unpublished data), while characterization of the UPy-aggregates in PBS was performed with optimized concentration of 50 μM. The UPy-monomers dissolved in methanol solutions were thoroughly mixed at required ratios (Table S1, ESI†), followed by adding phosphate buffered saline (PBS) or 4-(2-hydroxyethyl)-1-piperazineethanesulfonic acid (HEPES) (5 mM, pH 7.4) buffer to reach an UPy-monomer concentration of 50 μM. The UPy-suspension was then equilibrated overnight with shaking at 200 rpm to form the UPy-aggregates.

The assembly of UPy-monomers in PBS was characterized by examining the hydrophobic pocket formation at the lateral direction of the aggregates using Nile Red (NR) as a probe.^9^ The NR produces a hyperchromic effect upon encapsulation into a hydrophobic pocket.^47^ For all of the four types of systems (Fig. 1(b)), strong intensity of fluorescent NR signals were observed, which suggested the formation of hydrophobic domains in all cases (Fig. 1(c)). Besides, the wavelength of the emission spectra peaks for the systems of **neutral (+/−)**, **cationic**, **anionic** and **neutral** were 640, 637, 635 and 633 nm, respectively (Fig. 1(c)), which showed a blue shift. The shorter wavelength of the emission peaks indicated a more hydrophobic domain^48^ that represented a better fibrous assemblies formation. This blue shift suggests that the fibrous assemblies formation capability of the systems were in the order of **neutral** > **anionic** > **cationic** > **neutral (+/−)**. Dynamic light scattering (DLS) measurement showed that these UPy-aggregates had a hydrodynamic diameters between 50 and 300 nm (Fig. S1, ESI†). Fluorescence resonance energy transfer (FRET) experiments were performed by using the NR/Cy5 pair to confirm the incorporation of UPy–Cy5 into the UPy-aggregates, which is needed for monitoring intracellular uptake of the UPy-aggregates by fluorescence microscopy. Strong fluorescent signal of Cy5 was observed upon excitation of the encapsulated NR, which indicates the two fluorophores are in close proximity and the incorporation of UPy–Cy5 was successfully achieved (Fig. 1(d)).

The morphology of the UPy-aggregates containing 1 mol% UPy–Cy5 was then examined using the Total Internal Reflection Fluorescence (TIRF) microscope. Distinct UPy-aggregates were observed for all of the four systems (Fig. 1(e)). The **neutral** and **anionic** aggregates show long fibrous structures while their counterparts of **neutral (+/−)** and **cationic** aggregates are more rod-like particles (Fig. 1(e)). These results are in accordance with the NR measurements that also showed more blue shift of the wavelengths of the emission peaks for the **neutral** and **anionic** aggregates. Previously, it was reported that the incorporation of positively charged supramolecular monomers in the system impeded their growth and final polymer size.^49^ The rod-like structure formation of the **cationic** UPy-aggregates is proposed to be caused by the electrostatic repulsion of the protonated amine groups within a single supramolecular polymer. Although the micrograph shows fibrous structure for the **anionic** UPy-aggregates, its NR emission peak wavelength at 635 nm was longer than that of the **neutral** UPy-aggregates at 633 nm (Fig. 1(c) and (e)). This result also suggests that the charge repulsion between the deprotonated carboxylates impeded the supramolecular polymerization, which was also reported previously,^50^ although it was not as strong as their amine counterparts.

Zeta-potential of the UPy-aggregates were measured in 5 mM HEPES buffer at pH 7.4. The shift of the zeta-potential values of the four types of UPy-aggregates from negative to positive was observed, *i.e.*, −42.40 ± 8.57, −18.8 ± 5.76, −5.53 ± 3.70 and 33.6 ± 8.60 mV for **anionic**, **neutral (+/−)**, **neutral** and **cationic** UPy-aggregates, respectively (Fig. 1(f)). These results demonstrate that UPy-aggregates were successfully prepared with distinctive charge properties that correspond to the functional groups.

### Cytocompatibility of the UPy-aggregates

**Fig. 2:**
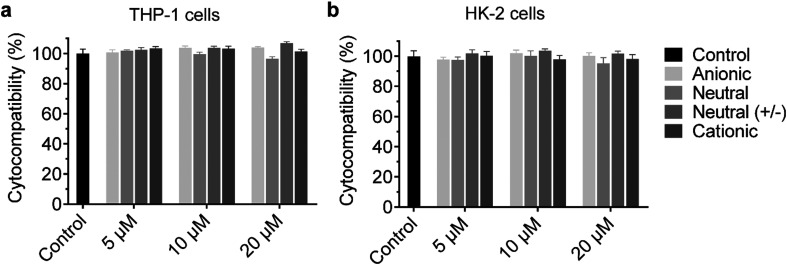
Cytocompatibility of the UPy-aggregates. The various UPy-aggregates all have good cytocompatibility with examined concentrations for both (a) THP-1 derived macrophages and (b) Human kidney cells (HK-2 cells). The tests were performed with an LDH method with the presence of fetal bovine serum, the cells without the addition of any UPy-aggregates were used as the control group. Error bars are the standard deviation of the mean value averaged over triplicates.

The **cationic** UPy-aggregates may bind to the membrane of the cells and disrupt the plasma membrane to induce cytotoxicity due to their positive charged property. With the disruption of the plasma membrane, the Lactate Dehydrogenase (LDH) in the cytoplasm is released and hence the cytotoxicity of the UPy-aggregates can be correlated to the damage of cell membranes.^51^ Therefore, the cytocompatibility of the UPy-aggregates was determined with an LDH test. To prepare the UPy-aggregates, the required amount of UPy-monomers in methanol solution were thoroughly mixed, followed by addition of cell culture medium containing 10% FBS and 1% antibiotics. The UPy-suspensions were then overnight equilibrated on a shaking bed at 200 rpm to form the UPy-aggregates with different concentrations. The LDH results showed that all four types of UPy-aggregates had good cytocompatibility for both THP-1 derived macrophages and HK-2 cells from 5 to 20 μM (Fig. 2). Although previous studies suggested that the positive charge property of nanocarriers are beneficial for their cellular uptake, this charge property may induce cytotoxicity.^42^ The UPy-aggregates used in the present study, however, did not show cytotoxicity even at a concentration of 20 μM.

### Influence of serum proteins and charge on the internalization of the UPy-aggregates

**Fig. 3:**
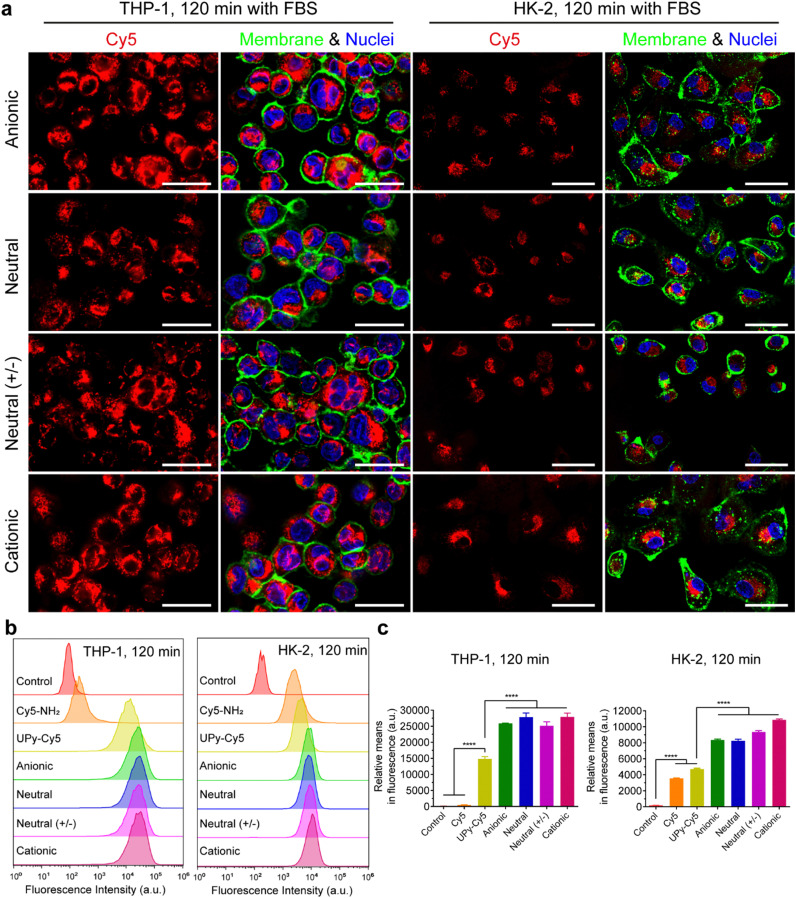
Internalization of UPy-aggregates (10 μM) by THP-1 derived macrophages and human kidney cells (HK-2) in the presence of fetal bovine serum (FBS). (a) Confocal laser scanning micrographs of the internalization of the UPy-aggregates at 120 min. The UPy-aggregates, nuclei and membranes of the cells were labeled red, blue and green, respectively. All of the UPy-aggregates with different charge properties were internalized by both THP-1 and HK-2 cells. Scale bars represent 30 and 50 μm for THP-1 and HK-2 cells, respectively. (b) Flow cytometry analysis of the internalization of the UPy-aggregates by THP-1 derived macrophages and HK-2 cells. The results are illustrated as the signal distribution of the 10 000 gated cells. The experimental group without addition of UPy-aggregates was used as the control. Both the internalizations of pure UPy–Cy5 and Cy5–NH_2_ were compared with the other four types of UPy-aggregates containing the same amount of Cy5, and much stronger fluorescence intensity for the cells of the four types of UPy-aggregates can be observed. (c) Illustration of the average fluorescence intensity of the 10 000 gated cells incubated with different materials. Error bars are the standard deviation of the mean cell fluorescence intensity averaged over triplicates. *p* < 0.0001 (****).

It is hypothesized that serum proteins strongly affect the charge influence of UPy-aggregates on cellular uptake. To test this hypothesis, the internalization of the UPy-aggregates by both THP-1 derived macrophages and HK-2 cells was qualitatively and quantitatively examined by live confocal imaging and flow cytometry, respectively. With FBS in the medium, the confocal live imaging results showed that UPy-aggregates were internalized by THP-1 derived macrophages in 5 min (Fig. S2a, ESI†). More aggregates were internalized with increasing incubation time until 120 min (Fig. 3(a) and Fig. S2a, ESI†). No significant differences, however, could be observed among the UPy-aggregates with different charge properties (Fig. 3(a) and Fig. S2a, ESI†). In contrast, the UPy-aggregates were internalized by HK-2 cells in a slower manner with visible cellular uptake after 30 min (Fig. S3a, ESI†). No significant differences could be observed among different charged UPy-aggregates at 120 min (Fig. 3(a)).

Flow cytometry results showed that both pure UPy–Cy5 and Cy5–NH_2_ were internalized by HK-2 cells (Fig. 3(b), (c) and Fig. S3b, c, ESI†). In contrast, only UPy–Cy5 was taken up by THP-1 derived macrophages even up to 120 min (Fig. 3(b), (c) and Fig. S2b, c, ESI†). Although pure UPy–Cy5 could be internalized by cells, the amount of internalized UPy–Cy5 as quantified from the fluorescent signal was much less compared to the fluorescent signal of the UPy-aggregates containing the same amount of UPy–Cy5 (Fig. 3(b), (c) and Fig. S2b, c, S3b, c, ESI†). These results suggest that the existence of UPy-aggregates stimulated their cellular internalization by both types of cells. Moreover, no significant difference on cellular internalization was observed among the UPy-aggregates with different charge properties upon 120 min incubation with cells (Fig. 3(b) and (c)). These results are in consistence with the results of live confocal imaging.

**Fig. 4:**
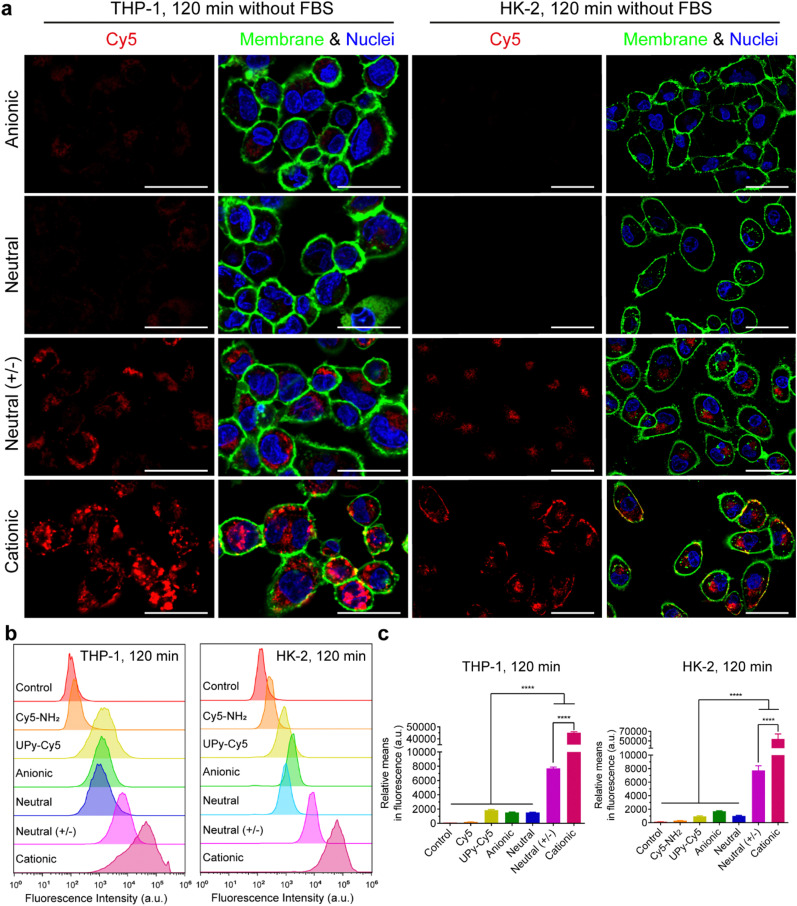
Internalization of the UPy-aggregates (10 μM) by THP-1 derived macrophages and human kidney cells (HK-2) in the absence of fetal bovine serum (FBS). (a) Confocal laser scanning micrographs of the internalization of UPy-aggregates at 120 min. The UPy-aggregates, nuclei and membranes of the cells were labeled red, blue and green, respectively. Both **neutral** and **anionic** UPy-aggregates were much less internalized compared to **cationic** and **neutral (+/−)** counterparts, which indicates the internalization of UPy-aggregates without the presence of FBS is charge dominated. Scale bars represent 30 and 50 μm for THP-1 derived macrophages and HK-2 cells, respectively. (b) Flow cytometry analysis of the internalization of the UPy-aggregates by THP-1 and HK-2 cells. The results are illustrated as the signal distribution of the 10 000 gated cells. The experimental group without addition of UPy-aggregates was used as the control. Both the internalizations of pure UPy–Cy5 and Cy5–NH_2_ were compared with the other four types of UPy-aggregates containing the same amount of Cy5, and much stronger fluorescence intensity for the cells of the **cationic** and **neutral (+/−)** groups of UPy-aggregates can be observed. (c) Illustration of the average fluorescence intensity of the 10 000 gated cells incubated with different materials. Error bars are the standard deviation of the mean cell fluorescence intensity averaged over triplicates. *p* < 0.0001 (****).

In the absence of FBS in the medium, live confocal imaging results showed that both the **cationic** and **neutral (+/−)** UPy-aggregates were internalized by THP-1 derived macrophages and HK-2 cells, with significantly more of the **cationic** UPy-aggregates being internalized compared to the **neutral (+/−)** aggregates (Fig. 4(a)). The **anionic** and **neutral** UPy-aggregates, however, were not taken up by both types of cells since no visible signal could be observed (Fig. 4(a)). Identical results were obtained from the flow cytometry experiments, with significant stronger fluorescence intensity and relative mean fluorescence intensity per cell for the **cationic** and **neutral (+/−)** UPy-aggregates compared to their **anionic** and **neutral** counterparts (Fig. 4(b) and (c)). Besides, the **cationic** UPy-aggregates with a higher positive charge were significantly more internalized than the **neutral (+/−)** UPy-aggregates (Fig. 4(b) and (c)). These results show that the internalization of UPy-aggregates by both THP-1 cells and HK-2 cells are charge dominated in the absence of serum proteins. Importantly, in this condition the cellular internalization of positively charged UPy-aggregates was enhanced.

In conclusion, the presence of serum proteins stimulated the cellular uptake of supramolecular UPy-aggregates for both phagocytic and non-phagocytic cells irrespective their charge properties (Fig. 3(a)). These results supported our hypothesis that the serum proteins overrule the influence of charge on cellular internalization of supramolecular nanostructures, although it is well recognized that the cationic nanocarriers are more favourable for cellular internalization.^39^ Moreover, these results indicate that the serum proteins may strongly interact with the UPy-aggregates, thus influence their cellular uptake and possibly their cellular locations.

### Location of the UPy-aggregates in cells

Live confocal images showed no overlap of fluorescent signals between Cy5 and membrane staining for THP-1 derived macrophages in all of these internalization studies (Fig. 3(a), 4(a) and Fig. S2a, ESI†). However, overlap of the fluorescent signals from Cy5 and membrane staining of HK-2 cells on the internalization of cationic UPy-aggregates in the absence of serum proteins was observed (Fig. 4(a)). This overlap of signals indicates the binding of positively charged UPy-aggregates onto the membranes of HK-2 cells.

Previous studies reported that Cy5-labeled oligonucleotides could colocalize with mitochondria after cellular internalization.^52,53^ The high magnification confocal micrographs of the internalized UPy-aggregates with different charge properties, which were prepared in the presence of serum proteins, showed rod-like morphology around the nuclei of cells from the fluorescent signal of Cy5 (Fig. S4, ESI†). This rod-like morphology is identical to the structure of mitochondria. Therefore, the mitochondria of the cells were labelled with green fluorescent proteins (GFP), which did not interfere with the surface properties of mitochondria.^54^ The locations of the UPy-aggregates in the cells were determined by using live confocal imaging.

**Fig. 5:**
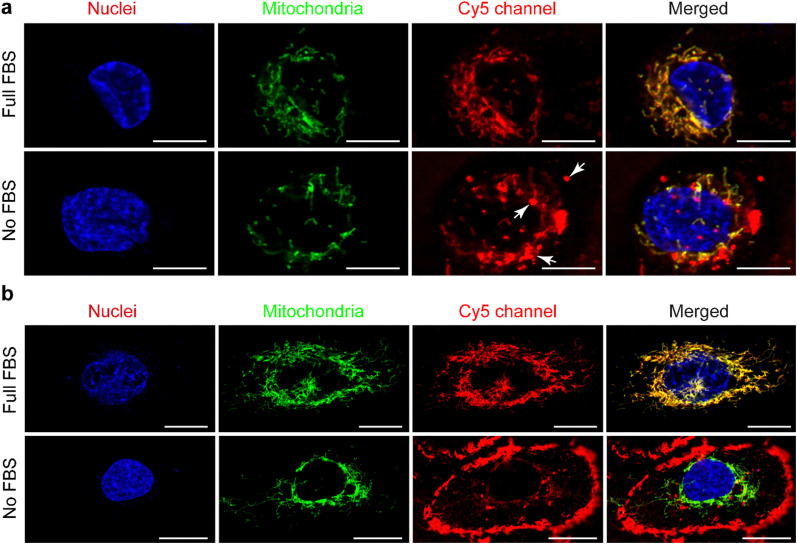
Locations of the **cationic** UPy-aggregates (10 μM) after being incubated with mammalian cells for 120 min. The **cationic** UPy-aggregates, GFP-modified mitochondria and nuclei of the cells were labelled red, green and blue, respectively. (a) The **cationic** UPy-aggregates can be internalized by THP-1 derived macrophages under the conditions of presence or absence of fetal bovine serum (FBS), and the Cy5 signal overlapped with mitochondria. Scale bars represent 10 μm. (b) The **cationic** UPy-aggregates can be internalized by human kidney cells (HK-2 cells) under the condition of presence of FBS, and the Cy5 signal overlapped with mitochondria. Under the condition of absence of FBS, the **cationic** UPy-aggregates anchored on the membranes of the HK-2 cells with subtle internalization. Scale bars represent 20 μm.

In the presence of FBS, the internalized **cationic** UPy-aggregates were co-localized with mitochondria for both THP-1 derived macrophages (Fig. 5(a)) and HK-2 cells (Fig. 5(b)), since the fluorescent signal of Cy5 overlapped with the GFP of mitochondria. Lacroix *et al.* also reported identical results for Cy5- and Cy3-labeled DNA nanostructures or oligonucleotides^55,56^ with both a cervical cancer cell line HeLa cells and a human liver cancer cell line HepG2 cells. In the absence of serum proteins, however, the location of the internalized **cationic** UPy-aggregates were significantly different for THP-1 derived macrophages and HK-2 cells. After internalization by THP-1 derived macrophages, the fluorescent Cy5 signal of the UPy-aggregates partly overlapped with the fluorescent signal of mitochondria while other parts of the Cy5 signal were displayed as vesicular structures (white arrows in Fig. 5(a)). These vesicular structures are proposed to be the phagosomes or endocytic vesicles containing internalized UPy-aggregates that were formed through phagocytosis (UPy-aggregates size > 0.5 μm) or endocytosis (UPy-aggregates size < 0.5 μm), respectively.^57,58^ For HK-2 cells, however, most of the UPy-aggregates were present at the surface of the cell membrane with minor internalization (Fig. 5(b)). These results indicate that the uptake and biodistribution of the UPy-aggregates by both THP-1 derived macrophages and HK-2 cells were dictated by serum proteins.

To further determine if these interactions between **cationic** UPy-aggregates and HK-2 cells were influenced by the molecular structure of the fluorescent dye, UPy-Fluorescein piperazine (UPy-F) was prepared and used as a fluorescent reporter to repeat the cellular internalization experiments. Identically, the **cationic** UPy-aggregates containing UPy-F prepared in the absence of serum protein presented mainly at the membranes of HK-2 cells, while they were mostly internalized by cells other than binding to membranes in the presence of serum protein (Fig. S5, ESI†). These results suggest that the internalization behaviour of UPy-aggregates into HK-2 cells was not likely influenced by the molecular nature of the fluorescent probes.

To further explore the influence of FBS on the location of UPy-aggregates after interacting with HK-2 cells, the HK-2 cells were incubated with the **cationic** UPy-aggregates in the absence of FBS. After 120 min incubation, the UPy-aggregates were washed away and the Dulbecco's Modified Eagle Medium (DMEM) containing full FBS was added to incubate the cells for another 120 min. Live confocal imaging results showed that part of the UPy-aggregates were internalized and co-localized with mitochondria, while part of aggregates anchored at the membranes of the cells (Fig. S6, ESI†). This result suggests that FBS can stimulate the internalization of **cationic** UPy-aggregates by HK-2 cells. Conclusively, the internalization process of amphiphilic **cationic** UPy-aggregates by HK-2 cells and the location of the aggregates were strongly influenced by serum proteins.

### Serum albumin regulates the cellular internalization of the UPy-aggregates

Serum albumin is the most abundant proteins in FBS, as well as in the bloodstream. It can bind to lipophilic molecules and assist their transport into the cells.^59,60^ In the present study, it was hypothesized that bovine serum albumin (BSA) presents in the FBS dominates the internalization of UPy-aggregates into HK-2 cells.

**Fig. 6:**
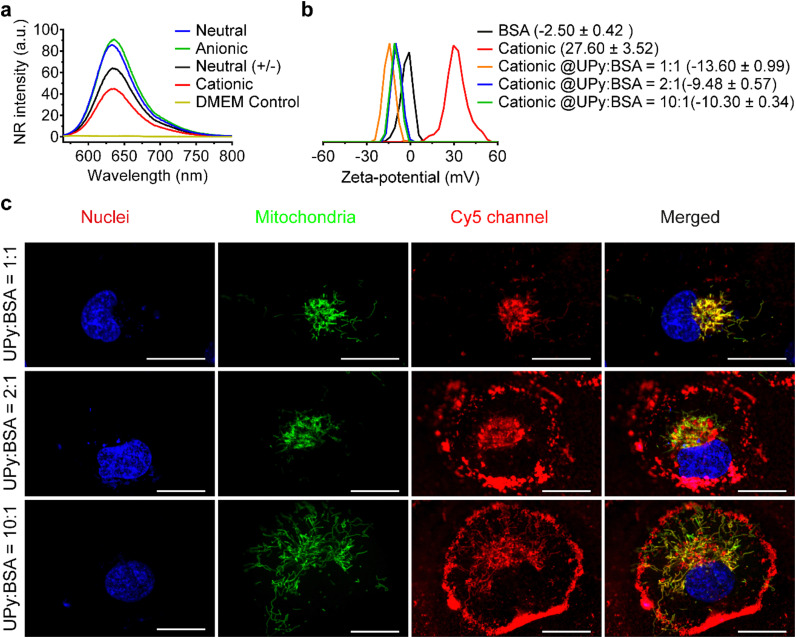
Bovine serum albumin (BSA) determined the internalization of **cationic** UPy-aggregates (10 μM) by human kidney cells. (a) Nile red (NR) test for the confirmation of the formation of UPy-aggregates in the DMEM medium with the formation of lateral hydrophobic pockets, where the NR could be encapsulated and emitted intensive fluorescent signals. The DMEM medium without addition of UPy-monomers was used as the control. (b) Zeta-potential of the **cationic** UPy-aggregates (50 μM) incubated with different amount of BSA in the medium of 5 mM HEPES buffer at pH 7.4. BSA strongly interacted with the **cationic** UPy-aggregates as shown by the zeta-potential shifting from positive to negative with different amount of BSA. (c) Confocal fluorescent micrographs of the internalization of the **cationic** UPy-aggregates with different amount of BSA. The **cationic** UPy-aggregates, GFP-modified mitochondria and nuclei of the cells were labeled red, green and blue, respectively. Scale bars represent 20 μm. With the addition of BSA into the **cationic** UPy-aggregates, the UPy-aggregates were gradually internalized by the cells and co-localized with mitochondria instead of anchoring on the membrane of the cells.

The formation of UPy-aggregates in the DMEM medium without BSA was examined. The results of the NR assay showed that all of the four types of UPy-aggregates could be formed in the DMEM medium since the samples containing UPy-monomers showed an increased NR intensity compared to the pure DMEM medium (Fig. 6(a)). Besides, the NR signal intensity of these UPy-aggregates was identical to those formed in the PBS (Fig. 1(c)), which suggests that the medium composition did not have significant influence on the formation of these UPy-aggregates. The zeta-potential of UPy-aggregates formed in 5 mM HEPES buffer (pH 7.4) with BSA at different UPy : BSA molar ratios was examined. The **cationic** UPy-aggregates became negatively charged with the addition of BSA into HEPES buffer containing UPy-monomers (Fig. 6(b)). These results show that BSA interacted strongly with these UPy-aggregates. Moreover, identical zeta-potential values of UPy-aggregates formed at different molar ratios of UPy : BSA were observed (Fig. 6(b)). Similar interactions between UPy-aggregates and BSA also occurred for the other UPy-aggregates with different charge properties (Fig. S7, ESI†).

Finally, the internalization of the UPy-aggregates in DMEM medium containing BSA with UPy : BSA at different molar ratios was monitored to confirm the function of BSA during cellular internalization process. With molar ratio of UPy : BSA = 10 : 1, only small amount the formed **cationic** UPy-aggregates were internalized by HK-2 cells and they co-localized with mitochondria (Fig. 6(c)). Most of the aggregates, however, remain adhered onto the membrane of the cells. With increasing the amount of BSA, gradually more of the **cationic** UPy-aggregates were internalized and less of the aggregates bounded onto the membrane (Fig. 6(c)). At the molar ratio of UPy : BSA = 1 : 1, the **cationic** UPy-aggregates were internalized by HK-2 cells and no membrane binding of these aggregates was observed (Fig. 6(c)). These results demonstrate that BSA played a strong regulatory role during the internalization of the **cationic** UPy-aggregates by HK-2 cells. With sufficient amount of BSA, which was at the molar ratio of UPy : BSA = 1 : 1, the aggregates can strongly interact with BSA to assist their intracellular uptake other than bind onto the membrane of the cells.

Although the morphology change of the UPy-aggregates influences their cellular internalization in the presence of BSA, the contribution of BSA on cellular internalization cannot be ruled out. Besides, other factors such as the density of PEG on the UPy-aggregates also have an impact on their interactions with BSA^61,62^ to influence cellular internalization. Therefore, further systematical investigation on the interaction between various types of UPy-aggregates and BSA, and the mechanism of BSA-regulated internalization are beneficial for the development of UPy-based supramolecular nanostructures for intracellular delivery.

## Experimental methods

### Materials

All solvents were purchased from commercial sources and used as received unless stated otherwise. Hoechst 33342, Invitrogen™ Live Cell Imaging Solution, CellMask™ Green plasma membrane stain, CellLight™ Mitochondria-GFP with BacMam 2.0 were purchased from Thermo Fisher Scientific. Phorbol-12-myristate-13-acetate (PMA) was ordered from Sigma-Aldrich.

### Synthesis

UPy–OCH_3_ was synthesized as described previously,^63^ with LC–MS [M] calculated *m*/*z* 1035.67, found 518.92 [M + 2H]^2+^ and 1036.33 [M + H]^+^. UPy–NH_2_ and UPy–COOH were synthesized as described previously,^64^ for UPy–NH_2_ with LC–MS [M] calculated *m*/*z* 1064.69, found 533.50 [M + 2H]^2+^ and 1065.58 [M + H]^+^, for UPy–COOH with [M] calculated *m*/*z* 1137.70, found 570 [M + 2H]^2+^ and 1138.67 [M + H]^+^. UPy–Cy5 and UPy-F were synthesized as described in ESI,† with LC–MS [M] of UPy–Cy5 calculated *m*/*z* 1701.11, found 567.75 [M + 3H]^3+^, 851.06 [M + 2H]^2+^ and 1701.33 [M + H]^+^; with LC–MS [M] of UPy-F calculated *m*/*z* 1519.82, found 1519.42 [M + H]^+^.

### Preparation of UPy aggregates

UPy-monomers were dissolved in methanol with a concentration of 2 mM, except for UPy–Cy5 and UPy-F at the concentration of 0.1 and 0.4 mM, respectively. To prepare aggregates with different charge properties (Table S1, ESI†), a certain amount of monomers were thoroughly mixed, followed by addition of PBS or HEPES to reach a UPy-monomer concentration of 50 μM, the UPy-suspensions were then equilibrated overnight under gentle shaking at 200 rpm. For cell internalization purpose, the UPy-aggregates (10 μM) were prepared by mixing required amount of monomer solutions, followed by addition of the culture medium containing 1% penicillin/streptomycin, with or without 10% FBS and equilibrated overnight or three hours on a shaking bed at 200 rpm, respectively.

### Characterizations of UPy-aggregates

Formation of the UPy-aggregates in PBS or DMEM medium was examined by a NR encapsulation test. In detail, NR was added into the 50 μM UPy-aggregates suspension to reach a concentration of 5 μM, with the UPy-monomer : NR molar ratio of 10 : 1. The suspension was then equilibrated by shaking at 150 rpm for 5 min. The emission spectrum was recorded from 565 nm to 800 nm using an excitation wavelength of 550 nm on a Varian Cary Eclipse fluorescence spectrometer (Agilent Technologies) using Quartz cuvettes. Encapsulation of Cy5 into the UPy-aggregates was examined by FRET measurement using the NR/Cy5 pair. The UPy–Cy5 was mixed with UPy-monomers at a molar ratio of 1 : 100 to reach the final concentrations of 0.5 μM and 50 μM, respectively. After formation of the UPy-aggregates, NR was then added to reach a concentration of 5 μM. After equilibrating, the emission spectrum was recorded from 540 to 800 nm using an excitation wavelength of 520 nm on the Varian Cary Eclipse fluorescence spectrometer. For all of these measurements, data of five scans were collected and averaged.

Total Internal Reflection Fluorescence (TIRF) images of the UPy-aggregates were acquired with a Nikon N-STORM system. The UPy-aggregates were prepared at 50 μM containing 1% UPy–Cy5 in PBS and diluted to 2 μM with PBS, followed by flowing in a chamber between a glass slide and microscope coverslip (Menzel-Gläser, #1, 24 × 24 mm) which were separated by a piece of double-sided tape. The sample was annealed for 2 min and subsequently washed twice with PBS. The Cy5 was excited at 647 nm and the fluorescence was filtered through a Nikon 97335 quad-band pass dichroic filter. The fluorescence was observed with a Nikon 100 × objective (1.4 NA oil immersion) and the images were recorded with an EMCCD camera (ixon3, Andor with pixel size 0.165 μm).

Due to the polydisperse properties of the UPy-aggregates, size and size distribution of the UPy-aggregates were measured with multi-angle Dynamic Light Scattering (DLS) with cumulant analysis.^65^ The UPy-aggregates were prepared at 50 μM in PBS and measured with a range of angles between 45° and 150° at an interval of 15°. Each angle was measured for 60 s with triplets at 20 °C. The hydrodynamic diameters of the UPy-aggregates were analyzed *via* cumulant analysis to obtain the *z*-average size by considering the UPy-aggregates as particles. Zeta-potential of the UPy-aggregates with a concentration of 50 μM in HEPES buffer (5 mM, pH 7.4) was measured by Laser Doppler Electrophoresis with a Malvern Zetasizer Nano-Z instrument (Worcestershire, UK) using a folded capillary cell (DTS 1060).

### Cell culture

THP-1 human monocytic cells and HK-2 cells were purchased from ATCC and cultured at 37 °C in 95% air/5% CO_2_ atmosphere with DMEM and Gibco™ Roswell Park Memorial Institute (RPMI) 1640 medium, respectively, supplemented with 10% FBS and 1% penicillin/streptomycin (P/S). THP-1 cells in suspension culture were passed every other day while the HK-2 cells were passed twice per week. For confocal fluorescence live imaging experiments and cytocompatibility measurement, the THP-1 derived macrophages and HK-2 cells were seeded at the density of 2.5 × 10^5^ and 2.5 × 10^4^ cells per cm^2^, respectively. To induce the differentiation of THP-1 monocytes into macrophages, PMA was added into the culture medium (50 ng mL^−1^) and incubated for 48 h.

### Cytocompatibility of the UPy-aggregates

Cytocompatibility of the various charged UPy-aggregates for both THP-1 derived macrophages and HK-2 cells was investigated with a LDH assay following the standard protocol (Thermo Fisher Scientific). Briefly, the cells were seeded onto a 48-well plate with the above mentioned density (*n* = 3). After attachment of the cells, the cell culture medium containing UPy-aggregates at various concentrations was then added into each well. The wells containing only culture medium were used as control. After 24 hours of incubation, lysis buffer was added into wells without UPy-aggregates and incubated for 45 min at 37 °C in 95% air/5% CO_2_ atmosphere, these wells were used as Maximum LDH. After this, a 50 μL aliquot of metabolic medium of each well was transferred into a 96-well flat-bottom plate with duplicates, followed by the addition of 50 μL of reaction mixture. The plate was then incubated at room temperature for 30 min protected from light. After incubation, 50 μL of stop solution was added into each well and the absorbance at 490 nm and 680 nm was measured at a plate reader. The LDH absorbance value was obtained by subtracting the 680 nm absorbance value (background) from the 490 nm absorbance value. The cytocompatibility of the UPy-aggregates was calculated with the follow equation:
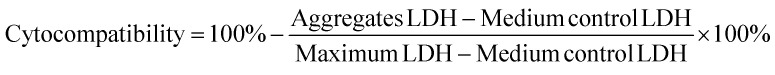


### Confocal fluorescence microscope

Different types of UPy-aggregates (Table S1, ESI† 10 μM) containing one percent of UPy–Cy5 as a reporter were prepared in DMEM and RPMI medium accordingly. THP-1 and HK-2 cells were seeded in an 8-well Thermo Fisher Scientific™ Nunc™ Lab-Tek™ Chamber with #1 borosilicate glass (*n* = 4). After attachment, the cells were washed three times with PBS and 0.4 mL of UPy-aggregates suspension was added into each well. For the experiments in the presence of FBS, the UPy-aggregates were incubated with cells for 5, 30 and 120 min. For the studies in the absence of FBS, the UPy-aggregates were incubated with cells for 120 min. At each time point, the UPy-aggregates were washed away with PBS, followed by sequential staining with Hoechst 33342 and CellMask™ Green plasma membrane stain for nuclei and membranes, respectively. After staining, the cells were washed three times with PBS and Invitrogen™ Live Cell Imaging Solution was added into each well for live imaging. The live imaging was performed under a Leica SP5 CLSM at 37 °C.

### Flow cytometry experiment

The amount of internalized UPy-aggregates by THP-1 derived macrophages and HK-2 cells were quantified by flow cytometry on a FACS Aria III (BD Biosciences) machine equipped with a 70 μm nozzle. The UPy-aggregates (Fig. 1(b) and Table S1, ESI† 10 μM) containing one percent of UPy–Cy5 as reporter were prepared in DMEM and RPMI media. THP-1 monocytes were seeded at a density of 2 × 10^5^ cells per cm^2^ in the 24-well plates (*n* = 3) while HK-2 cells were seeded onto the 6-well plates with a density of 2 × 10^4^ cells per cm^2^ to avoid the overlap of the cells in the middle of each well (*n* = 3). After attachment, the cells were washed three times with PBS and incubated with UPy-aggregates for a certain period. The cells were then gently washed three times with PBS and trypsinized with trypsin for 2 min. Trypsin was deactivated by the addition of full medium and the cells were collected by centrifugation for 5 min at 150 and 130 g for THP-1 derived macrophages and HK-2 cells, respectively. The collected cell pellet was re-suspended in 500 μL of PBS containing 1 mg mL^−1^ BSA and loaded into the flow cytometry. A total amount of 10 000 single cells were counted and gated based on their forward *vs.* side scatter signals. The fluorescence intensity of per cell was measured by exciting the Cy5 in the UPy-aggregates with a 633 laser and detect through a 650/670 bandpass filter. All of the samples were measured with triplicate at a flow rate of 1 mL min^−1^.

### Determination of the location of internalized UPy-aggregates

CLSM live imaging with the mitochondria staining was performed to explore the location of the UPy-aggregates after incubation with cells. **Cationic** UPy-aggregates (Fig. 1(b) and Table S1, ESI† 10 μM) containing one percent of UPy–Cy5 as a reporter were prepared in DMEM medium in the presence or absence FBS. THP-1 and HK-2 cells were seeded in an 8-well Thermo Fisher Scientific™ Nunc™ Lab-Tek™ Chamber with #1 borosilicate glass bottom (*n* = 4). After attachment of the cells, 20 and 8 μL of CellLight® Mitochondria-GFP BacMam 2.0 Reagent were added into each well for THP-1 derived macrophages and HK-2 cells, respectively, to induce the mitochondria GFP secretion. The cells were then washed three times with PBS after overnight culture. The **cationic** UPy-aggregates (0.4 mL) in media with or without FBS were added and the cells were incubated for 120 min. After that, the cells were gently washed three times with PBS and stained by Hoechst 33324. Location of the **cationic** UPy-aggregates was then observed under CLSM live imaging at 37 °C.

### Determination of the influence of BSA on cellular uptake of UPy-aggregates

The cellular internalization of **cationic** UPy-aggregates with the presence of different amount of BSA was examined to determine the function of BSA on the cellular uptake of UPy-aggregates by HK-2 cells. The **cationic** UPy-aggregates (Fig. 1(b) and Table S1, ESI† 10 μM) containing one percent of UPy–Cy5 as a reporter were prepared in DMEM media containing different amounts of BSA. The HK-2 cells were seeded in an 8-well Thermo Fisher Scientific™ Nunc™ Lab-Tek™ Chamber with #1 borosilicate glass bottom (*n* = 4). After cell attachment, 8 μL of CellLight® Mitochondria-GFP BacMam 2.0 Reagent was added into each well with overnight incubation to induce the mitochondria GFP secretion. The cells were then washed three times with PBS and 0.4 mL of prepared UPy-aggregates suspension was added into each well for 120 min incubation. Subsequently, the cells were washed twice with PBS and the nuclei of the cells were stained with Hoechst. CLSM live imaging was performed to monitor the location of the UPy-aggregates in the cells.

## Conclusions

In conclusion, the influence of serum proteins on the internalization of various charged supramolecular polymer UPy-based nanostructures by phagocytic and non-phagocytic cells, *i.e.*, THP-1 derived macrophages and HK-2 cells, was systematically investigated. Serum proteins dictated the cellular uptake of UPy-aggregates irrespective their charge properties. Additionally, the internalized UPy-aggregates co-localized with mitochondria of the cells. In the absence of serum proteins, only positively charged UPy-aggregates could be internalized by THP-1 derived macrophages, while most of the positively charged UPy-aggregates anchored at the membranes of HK-2 cells other than being internalized. The UPy-aggregates interacted strongly with BSA and could be internalized by HK-2 cells with the presence of BSA. Therefore, we propose that BSA regulates the cellular internalization of UPy-aggregates. This study opens the door for the investigation of serum albumin on the internalization of supramolecular polymer assemblies and provides fundamental insights for the fabrication of supramolecular polymer nanostructures for intracellular delivery purposes.

## Author contributions

Jiankang Song: conceptualization, methodology, resources, investigation, data curation, validation, formal analysis, visualization, writing – original draft. Peter-Paul K. H. Fransen: resources, writing – review & editing. Maarten H. Bakker: resources, writing – review & editing. Sjors P. W. Wijnands: investigation, writing – review & editing. Jingyi Huang: resources, writing – review & editing. Shuaiqi Guo: investigation, writing – review & editing. Patricia Y. W. Dankers: funding acquisition, conceptualization, visualization, supervision, writing – review & editing. All authors have given approval to the final version of the manuscript.

## Conflicts of interest

There are no conflicts to declare.

## Supplementary Material

TB-012-D3TB02631K-s001
