# Supplementary material for: The effect of charge and albumin on cellular uptake of supramolecular polymer nanostructures
Source: J Mater Chem B. 2024 Apr 9;12(20):4854–66. doi: 10.1039/d3tb02631k (PMC11111113; doi:10.1039/d3tb02631k)
Supplement: TB-012-D3TB02631K-s001 [file TB-012-D3TB02631K-s001.pdf]

Electronic Supplementary Material (ESI) for Journal of Materials Chemistry B.
This journal is © The Royal Society of Chemistry 2024

Electronic Supplementary Information

The effect of charge and albumin on cellular uptake of supramolecular polymer nanostructures

Jiankang Song[1,2,3], Peter-Paul K.H. Fransen[1,3], Maarten H. Bakker[1,3], Sjors P.W. Wijnands[1,3], Jingyi Huang[1,3], Shuaiqi Guo[1] and Patricia Y.W. Dankers[1,2,3*]

[1]Institute for Complex Molecular Systems, Eindhoven University of Technology, the Netherlands

[2]Department of Biomedical Engineering, Laboratory for Cell and Tissue Engineering, Eindhoven University of Technology, the Netherlands

[3]Department of Biomedical Engineering, Laboratory of Chemical Biology, Eindhoven University of Technology, the Netherlands

†Corresponding author:

Patricia Y.W. Dankers

Institute for Complex Molecular Systems

Eindhoven University of Technology

P.O. Box 513, 5600 MB Eindhoven, The Netherlands

Tel.: +31 (0) 40 24 75 451

E-mail: P.Y.W.Dankers@tue.nl

Synthesis

Synthesis of UPy-Cy5

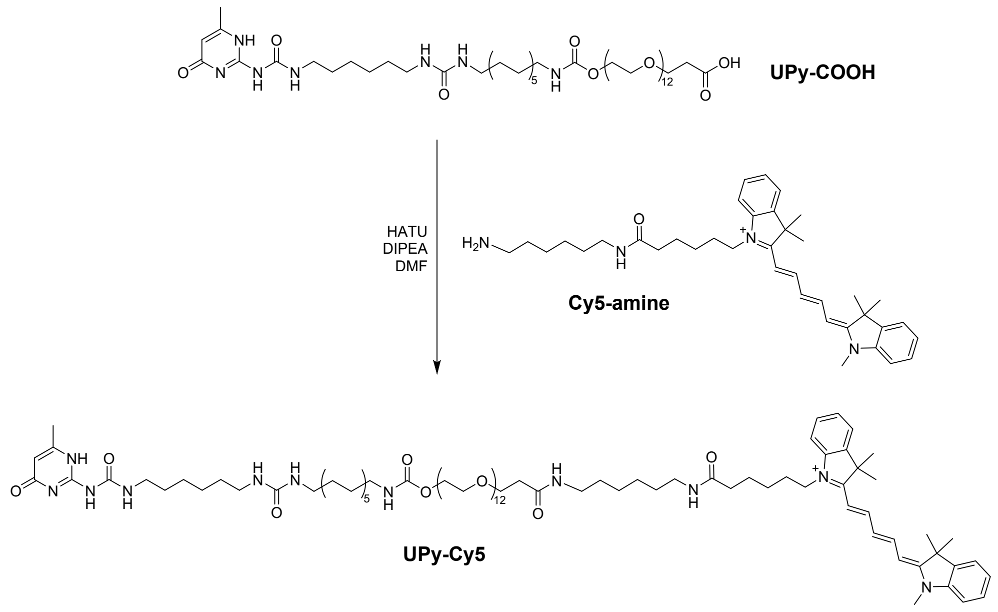

The deprotected **UPy-COOH** (12 mg; 0.011 mmol) was dissolved in 2 mL DMF. HATU (5 mg; 0.012 mmol) and DIPEA (9 µL; 0.048 mmol) were then added for pre-activation and the mixture was stirred for five minutes. After that, the **Cy5-amine** was added (5 mg; 0.0086 mmol) and the reaction was stirred for one hour. The organic phase was then diluted in DCM (10 mL), followed by washing with brine and evaporation of the DCM. The resulting crude was purified by preparative reverse phase LC-MS to yield the desired compound (5.8 mg; 31%). The LC-MS [M] of **UPy-Cy5** was calculated 1701.11, found 567.75 $[M+3H]^{3+}$, 851.06 $[M+2H]^{2+}$ and 1701.33 $[M+H]^{+}$.

Synthesis of UPy-F

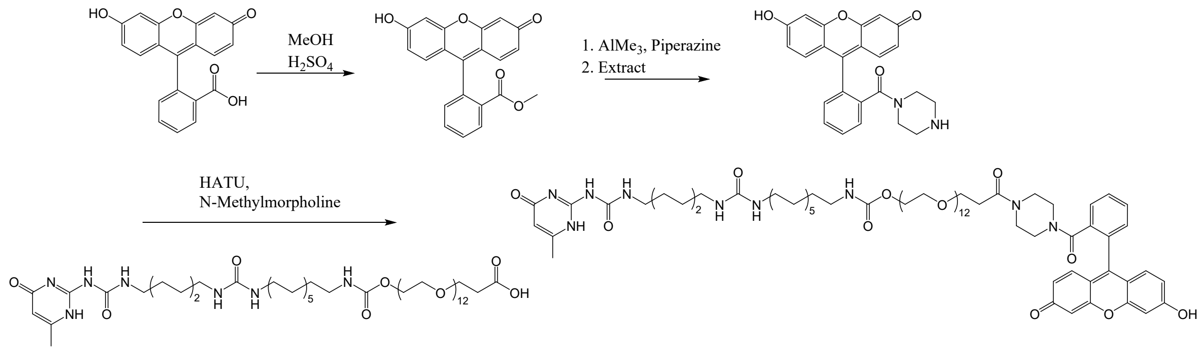

Fluorescein piperazine was prepared as described previously in literature.[1] The deprotected **UPy-COOH** (10 mg, 0.01 mmol) was dissolved in DMF (2 mL). HATU (5.1 mg, 0.13 mmol) and pyridine (0.11 mL, 0.13 mmol) were then added with stirring under argon for 30 minutes. Thereafter, 1 mL of Fluorescein piperazine (8 mg, 0.02 mmol) in DMF was added. The reaction mixture was stirred overnight and subsequently poured into 2 v/v% formic acid/water solution and centrifuged for two times. Eluting over silica with FA/MeOH/$CHCl_3$ 1:5:94 afforded UPy-fluorescein-piperazine (13.2 mg, 87%) as a yellow solid. The LC-MS MS [M] of UPy-F was calculated m/z 1519.82, found 1519.42 $[M+H]^+$.

Supporting data

Table S1. Composition of UPy-aggregates with different charge properties

Group Name	UPy-OCH$_3$	UPy-NH$_2$	UPy-COOH	Note
**Anionic**	50		50	
**Neutral**	100			All in molar
**Neutral (+/-)**		50	50	ratio
**Cationic**	50	50		

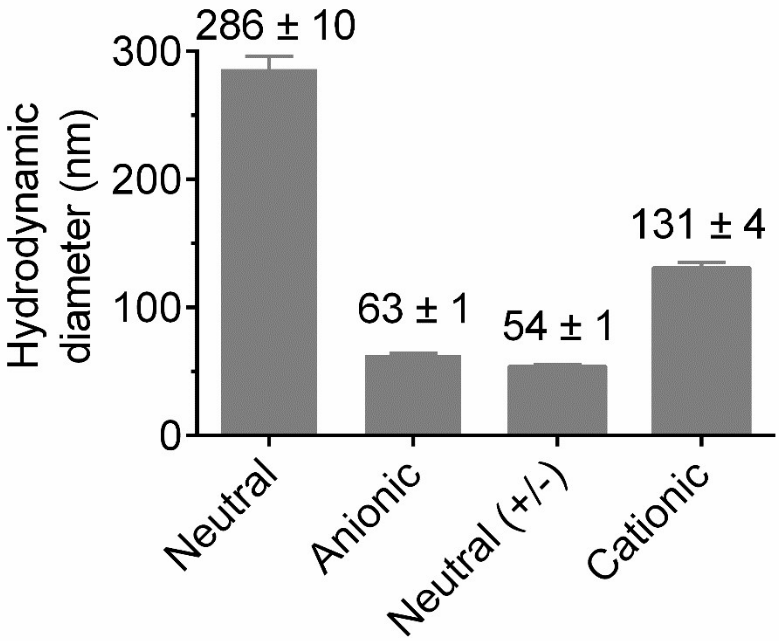

Figure S1: Sizes of the UPy-aggregates with various charge properties. The sizes of the UPy-aggregates were measured by multi-angle dynamic light scattering method. The results are illustrated as the hydrodynamic diameters of the UPy-aggregates which were obtained via cumulant analysis to obtain the z-average size by considering the UPy-aggregates as particles.

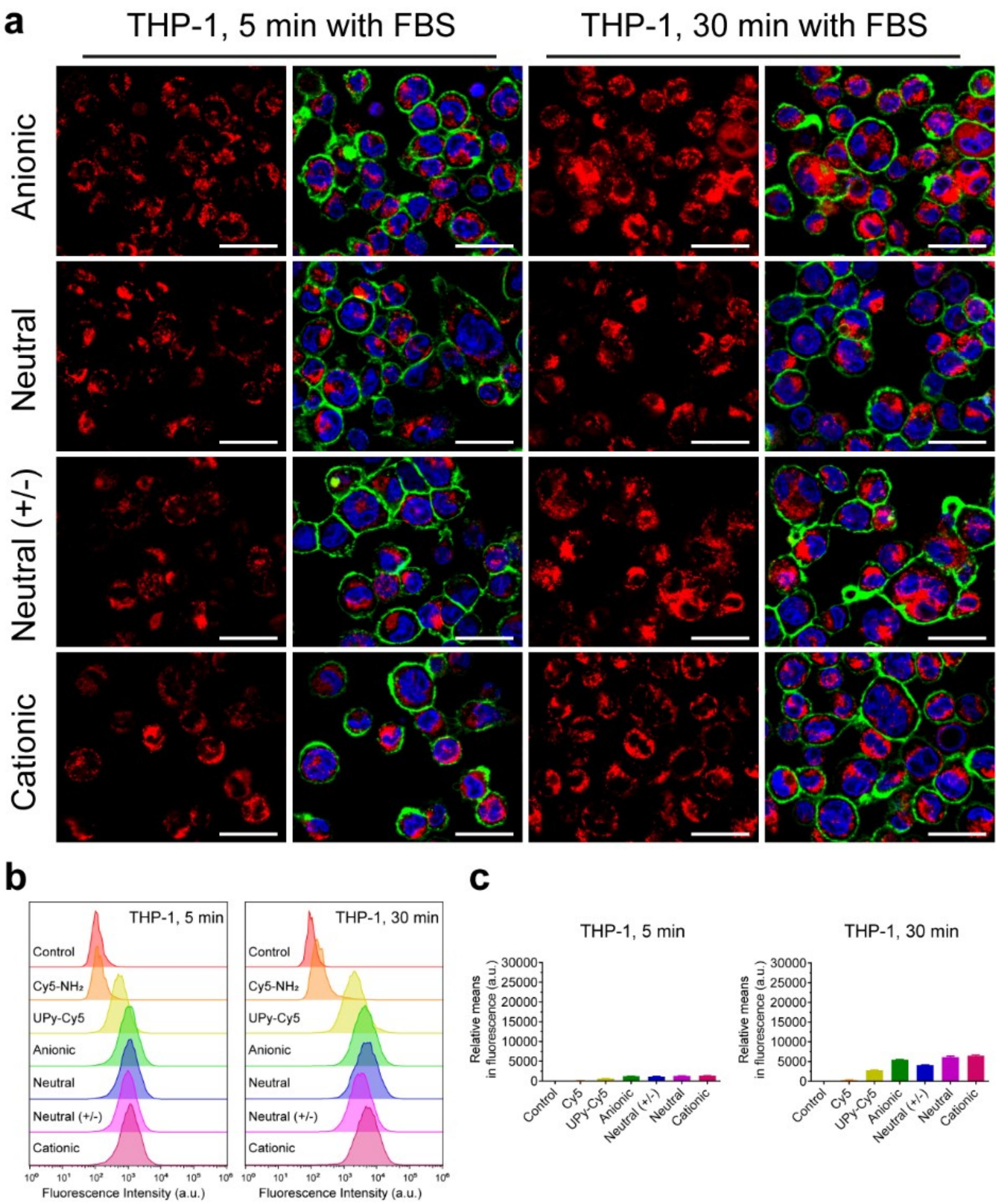

Figure S2: Internalization of UPy-aggregates (10 µM) by THP-1 derived macrophages in the presence of fetal bovine serum (FBS). (a) Confocal laser scanning micrographs of the internalization of the UPy-aggregates at 5 and 30 min. The UPy-aggregates, nuclei of the cells and membranes of the cells were labeled red, blue and green, respectively. Scale bars represent 30 µm. (b) Flow cytometry analysis of the internalization of the UPy-aggregates by

THP-1 cells at 5 and 30 min. The results are illustrated as the signal distribution of the 10,000 gated cells. The experimental group without addition of UPy-aggregates was used as the control. Both the internalizations of pure UPy-Cy5 and Cy5-NH_2 were compared with the other four types of UPy-aggregates containing the same amount of Cy5. (c) Illustration of the average fluorescence intensity of the 10,000 gated cells incubated with different materials. Error bars are the standard deviation of the mean cell fluorescence intensity averaged over triplicates.

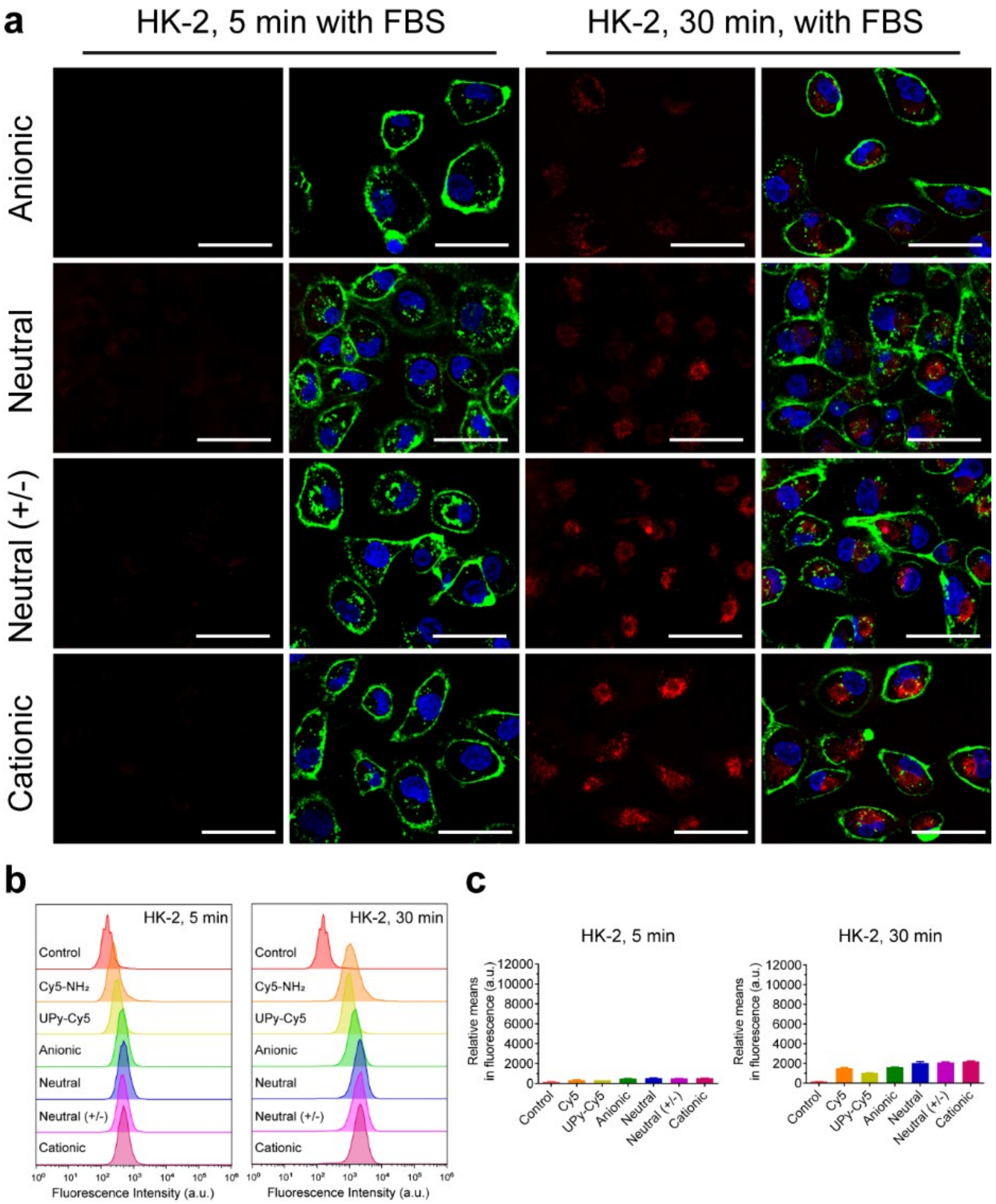

Figure S3: Internalization of UPy-aggregates (10 μM) by Human Kidney cells (HK-2) in the presence of fetal bovine serum (FBS). (a) Confocal laser scanning micrographs of the internalization of the UPy-aggregates at 5 and 30 min. The UPy-aggregates, nuclei of the cells and membranes of the cells were labeled red, blue and green, respectively. Scale bars represent 50 μm. (b) Flow cytometry analysis of the internalization of the UPy-aggregates by

HK-2 cells at 5 and 30 min. The results are illustrated as the signal distribution of the 10,000 gated cells. The experimental group without addition of UPy-aggregates was used as the control. Both the internalizations of pure UPy-Cy5 and Cy5-NH_2 were compared with the other four types of UPy-aggregates containing the same amount of Cy5. (c) Illustration of the average fluorescence intensity of the 10,000 gated cells incubated with different materials. Error bars are the standard deviation of the mean cell fluorescence intensity averaged over triplicates.

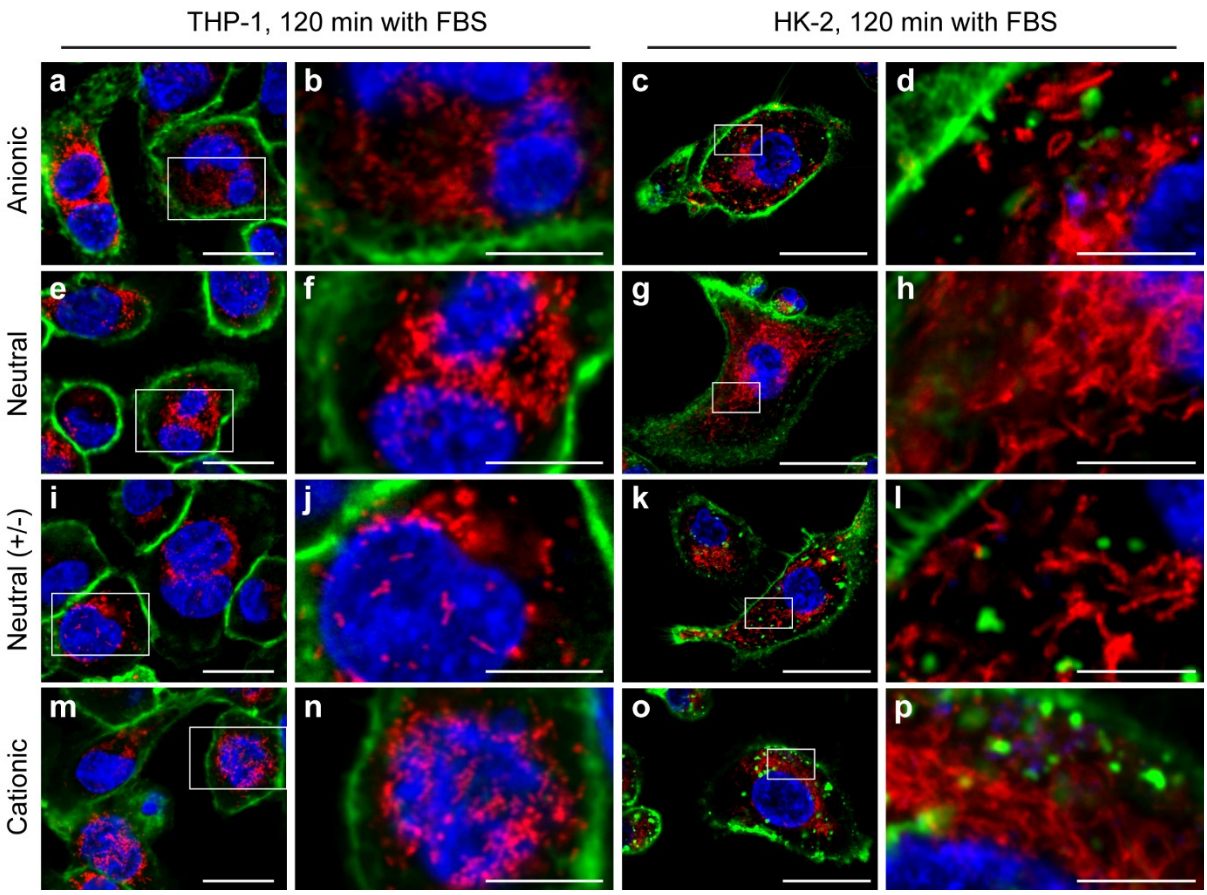

Figure S4: Distribution of UPy-aggregates (10 μM) after internalization by THP-1 derived macrophages and human kidney cells (HK-2) at 120 min in the of presence of fetal bovine serum (FBS). Confocal laser scanning micrographs of the cells incubated with UPy-aggregates **anionic** (a-d), **neutral** (e-h), **neutral (+/-)** (i-l) and **cationic** (m-p); b, f, j and n are the zoom-in pictures of the white frames in a, e, i and m, respectively; d, h, i and p are the zoom-in pictures of the white frames in c, g, k and o, respectively; The UPy-aggregates were labeled red, nuclei of the cells were stained blue and membranes of the cells were stained green; scale bars in a, e, i and m represent 20 μm, scale bars in b, f, j, n, d, h, i and p represent10 μm, scale bars in c, g, k and o represent 50 μm.

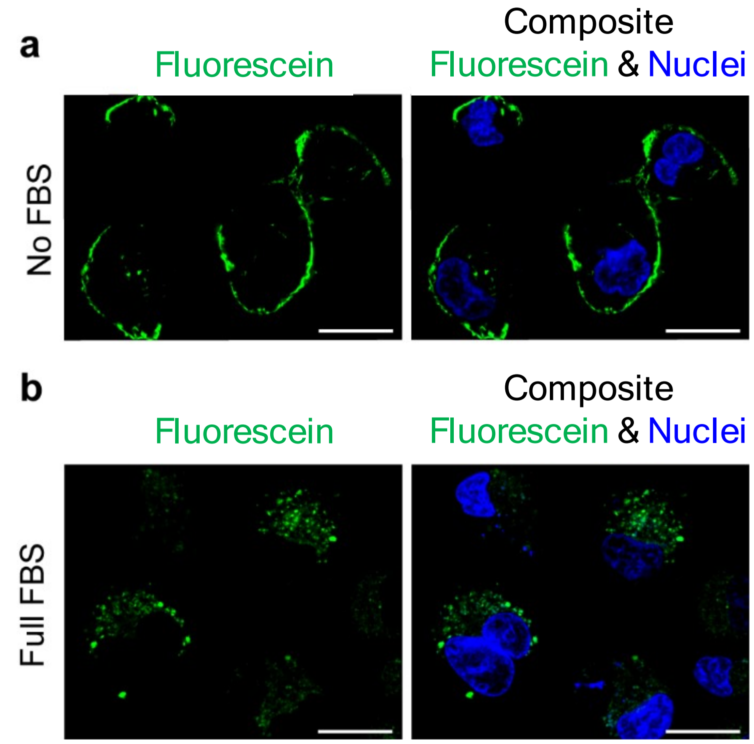

Figure S5: Confocal laser scanning micrographs of the internalization of **cationic** UPy-aggregates (10 µM) containing UPy-Fluorescein (UPy-F) (0.8 µM) by human kidney cells (HK-2). The nuclei of the cells were stained blue and the UPy-aggregates were stained green. The UPy-aggregates were incubated with HK-2 cells for 120 min, they localized mainly at the cell membranes in the absence of fetal bovine serum (FBS), while they were internalized by cells in the presence of FBS. Scale bars represent 20 µm.

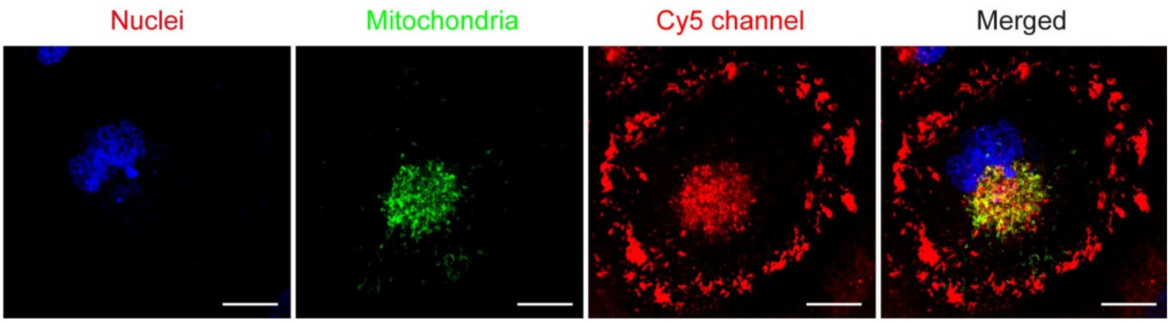

Figure S6: Confocal laser scanning micrographs of **cationic** UPy-aggregates internalized by human kidney cells (HK-2). The UPy-aggregates were formed in the DMEM without fetal bovine serum (FBS) for three hours and were then incubated with the HK-2 cells for 120 min at the concentration of 10 µM. After that, the aggregates were washed away and the cells were incubated with DMEM medium containing full FBS but without UPy-aggregates for 120 min. Scale bars represent 20 µm.

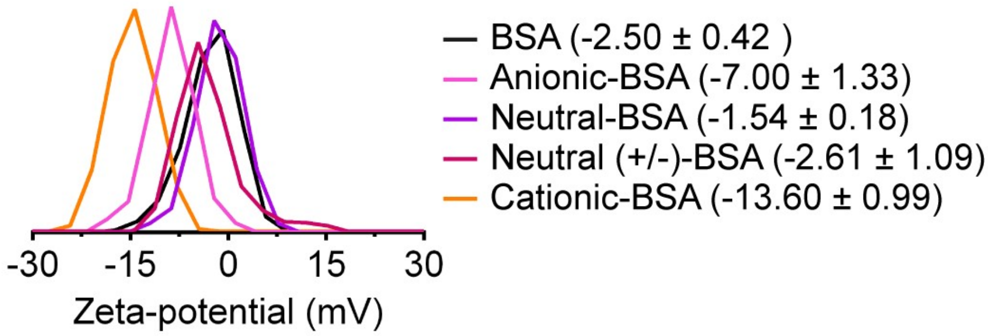

Figure S7: Zeta-potential of the UPy-aggregates with various charge properties incubated with the same amount of bovine serum albumin (BSA) in the medium of 5 mM HEPES buffer at pH 7.4. The concentration of both UPy and BSA were 50 µM.

Reference

1. Chang, P. V.; Prescher, J. A.; Hangauer, M. J.; Bertozzi, C. R., Imaging Cell Surface Glycans with Bioorthogonal Chemical Reporters. Journal of the American Chemical Society 2007, 129 (27), 8400-8401.
